# Absence of system x_c_^−^ on immune cells invading the central nervous system alleviates experimental autoimmune encephalitis

**DOI:** 10.1186/s12974-016-0787-0

**Published:** 2017-01-13

**Authors:** Ellen Merckx, Giulia Albertini, Magdalena Paterka, Cathy Jensen, Philipp Albrecht, Michael Dietrich, Joeri Van Liefferinge, Eduard Bentea, Lise Verbruggen, Thomas Demuyser, Lauren Deneyer, Jan Lewerenz, Geert van Loo, Jacques De Keyser, Hideyo Sato, Pamela Maher, Axel Methner, Ann Massie

**Affiliations:** 1 Center for Neurosciences (C4N), Department of Pharmaceutical Biotechnology and Molecular Biology, Vrije Universiteit Brussel, Laarbeeklaan 103, 1090 Brussels, Belgium; 2 Center for Neurosciences (C4N), Department of Pharmaceutical Chemistry and Drug Analysis, Vrije Universiteit Brussel, Brussels, Belgium; 3 Department of Neurology, University Medical Center of the Johannes Gutenberg-University of Mainz, Mainz, Germany; 4 Department of Neurology, Medical Faculty, Heinrich-Heine University, Düsseldorf, Germany; 5 Department of Neurology, University of Ulm, Ulm, Germany; 6 Inflammation Research Center, VIB and Department of Biomedical Molecular Biology, Ghent University, Ghent, Belgium; 7 Center for Neurosciences (C4N), Department of Neurology, Universitair Ziekenhuis Brussel, Brussels, Belgium; 8 Department of Medical Technology, Faculty of Medicine, Niigata University, Niigata, Japan; 9 Cellular Neurobiology Laboratory, Salk Institute for Biological Studies, La Jolla, CA USA

**Keywords:** System x_c_^−^, xCT, Glutamate, Multiple sclerosis, Experimental autoimmune encephalomyelitis

## Abstract

**Background:**

Multiple sclerosis (MS) is an autoimmune demyelinating disease that affects the central nervous system (CNS), leading to neurodegeneration and chronic disability. Accumulating evidence points to a key role for neuroinflammation, oxidative stress, and excitotoxicity in this degenerative process. System x_c_^−^ or the cystine/glutamate antiporter could tie these pathological mechanisms together: its activity is enhanced by reactive oxygen species and inflammatory stimuli, and its enhancement might lead to the release of toxic amounts of glutamate, thereby triggering excitotoxicity and neurodegeneration.

**Methods:**

Semi-quantitative Western blotting served to study protein expression of xCT, the specific subunit of system x_c_^−^, as well as of regulators of xCT transcription, in the normal appearing white matter (NAWM) of MS patients and in the CNS and spleen of mice exposed to experimental autoimmune encephalomyelitis (EAE), an accepted mouse model of MS. We next compared the clinical course of the EAE disease, the extent of demyelination, the infiltration of immune cells and microglial activation in xCT-knockout (xCT^−/−^) mice and irradiated mice reconstituted in xCT^−/−^ bone marrow (BM), to their proper wild type (xCT^+/+^) controls.

**Results:**

xCT protein expression levels were upregulated in the NAWM of MS patients and in the brain, spinal cord, and spleen of EAE mice. The pathways involved in this upregulation in NAWM of MS patients remain unresolved. Compared to xCT^+/+^ mice, xCT^−/−^ mice were equally susceptible to EAE, whereas mice transplanted with xCT^−/−^ BM, and as such only exhibiting loss of xCT in their immune cells, were less susceptible to EAE. In none of the above-described conditions, demyelination, microglial activation, or infiltration of immune cells were affected.

**Conclusions:**

Our findings demonstrate enhancement of xCT protein expression in MS pathology and suggest that system x_c_^−^ on immune cells invading the CNS participates to EAE. Since a total loss of system x_c_^−^ had no net beneficial effects, these results have important implications for targeting system x_c_^−^ for treatment of MS.

## Background

Multiple sclerosis (MS) is an autoimmune disease that affects about 2.5 million people worldwide. Myelin, the insulating sheath surrounding nerve cells, is attacked and impaired by infiltrating T cells and macrophages, ultimately leading to disturbed neural signaling and transmission by axons [1, 2]. It is believed that an imbalance in glutamate homeostasis together with oxidative stress contributes to neurodegeneration [3–6]. In addition, neuroinflammation is undeniably involved in the pathogenesis of chronic neurodegeneration in MS [7].

Glutamate, the most abundant excitatory neurotransmitter in the central nervous system (CNS), is released from presynaptic vesicles into the synaptic cleft where it activates pre- and postsynaptic receptors before being removed via uptake by excitatory amino acid transporters (high-affinity Na^+^/K^+^-dependent glutamate uptake transporter or excitatory amino acid transporters (EAATs)). Increased extracellular glutamate levels lead to massive stimulation of ionotropic glutamate receptors, a process known as excitotoxicity, and induce neuronal and oligodendrocytic cell death [4]. Excitotoxicity is involved in several neurodegenerative diseases [8], including MS [9]. Indeed, increased glutamate levels were observed in the cerebrospinal fluid and in normal appearing white matter (NAWM) of MS patients [10–13]. In search of the culprit for these elevated glutamate levels, glutamate-metabolizing enzymes (glutamine synthetase and glutamate dehydrogenase) [14–17] as well as glutamate receptors [18–23] and EAATs [24–28] have been extensively investigated in the brain tissue of MS patients and animal models, such as the experimental autoimmune encephalomyelitis (EAE) mouse model of inflammatory demyelination [29] and the Theiler’s murine encephalomyelitis virus-induced demyelination model [30].

The cystine/glutamate antiporter or system x_c_^−^ is a sodium-independent glutamate transporter that imports extracellular cystine in exchange for intracellular glutamate [31]. System x_c_^−^ is composed of the subunit 4F2 heavy chain and the specific light chain subunit xCT [32] and is a major source of extracellular glutamate in several rodent brain regions such as the striatum and hippocampus [33–35]. Cysteine, the reduced form of the imported cystine, is the rate-limiting amino acid necessary for the synthesis of the major antioxidant of the CNS, glutathione (GSH). Expression and activity of system x_c_^−^ are enhanced by reactive oxygen species (ROS) and inflammatory stimuli [36–38] and, besides GSH production, this increase can potentially lead to glutamate toxicity and neurodegeneration [39, 40].

Due to its potential relevance in neurological disorders [40], system x_c_^−^ has recently been studied in MS; nevertheless, the results are still controversial. Pampliega et al. showed enhanced xCT mRNA expression levels in leukocytes and postmortem optic nerve samples of MS patients compared to healthy controls. Additionally, increased xCT protein and mRNA expression was found in the spinal cord of rats exposed to EAE as compared to control rats [41]. Moreover, using positron emission tomography imaging with (4S)-4-(3-(18)F-fluoropropyl)-l-glutamate, a novel radiotracer to assess system x_c_^−^ activity, it was shown that its function was enhanced in the same model [42]. In C3H/HeSnJSlc7a11^sut/sut^ (sut/sut) mice that are deficient for system x_c_^−^, proteolipid protein (PLP)-induced EAE was attenuated compared to their wild type littermates. The latter effect could be replicated by pharmacological inhibition of system x_c_^−^ with sulfasalazine (SAS) and (S)-4-carboxyphenylglycine [43]. Whereas the above-described studies suggest that enhancement of system x_c_^−^ contributes to the pathogenesis of MS, a recent report of Morales Pantoja et al. showed diminished xCT mRNA and protein levels in the mouse spinal cord during the course of myelin oligodendrocyte glycoprotein (MOG)_35–55_ peptide-induced EAE [44]. Moreover, Soria et al. demonstrated that pharmacological inhibition of system x_c_^−^ with SAS-induced myelin damage and speculated that the cystine/glutamate antiporter is a key factor for the maintenance of oligodendrocyte homeostasis [45]. We here sought to shed light on this controversial subject and clarify the potential of system x_c_^−^ as a drug target in the treatment of MS, by investigating xCT protein expression levels in NAWM of MS patients as well as the CNS and spleen of EAE-induced mice and by exploring whether EAE development is affected by xCT deletion using xCT knockout (xCT^−/−^) mice with a C57BL/6J background [46] or bone marrow (BM) chimeras thereof.

We describe for the first time increased xCT protein expression in the NAWM of MS patients compared to controls without neurological disease. Moreover, we show that this xCT upregulation is independent of activating transcription factor 4 (ATF4), a transcription factor known to initiate transcription of xCT [38, 47, 48]. In agreement with our human data, xCT protein expression was augmented in the CNS as well as the spleen of EAE mice. xCT^−/−^ mice were equally susceptible to EAE than their wild-type (xCT^+/+^) littermates. However, the lack of xCT exclusively in immune cells attenuated EAE compared to control mice. We therefore hypothesize that system x_c_^−^ activity on immune cells invading the CNS might be involved in EAE- and MS-dependent neurodegeneration.

## Methods

### MS patients and controls

Freshly frozen brain tissue of MS patients and geographically matched controls has been collected from donors for or from whom a written informed consent for a brain autopsy and the use of the material and clinical information for research purposes had been obtained by the Human Brain and Spinal Fluid Resource Center in Los Angeles (USA). The frozen tissue was stored at −80 °C until use. The tissue was sectioned in 5 and 50 μm sections. NAWM was collected from the 50-μm brain slices and used to extract total RNA or protein. Adjacent 5 μm cryosections were assessed by a neuropathologist to confirm the localization of lesions and NAWM.

### Animals

All mice used in this study were 10–15-week-old females and were housed and maintained in accordance with national guidelines on animal experimentation. Experiments were approved by the ethical committee for animal experimentation of the Faculty of Medicine and Pharmacy of the Vrije Universiteit Brussel, of the Faculty of Sciences of Ghent University or of the University of Mainz. C57BL/6J mice were obtained from Charles River Laboratories (France). The results are presented in accordance with the ARRIVE guidelines for reporting experiments involving animals [49].

xCT^−/−^ mice and their xCT^+/+^ littermates have a C57BL/6J background and are high-generation descendants of the strain described previously [46]. Despite the absence of xCT, these mice are healthy and fertile [46]. They have unaffected hippocampal and striatal GSH levels and do not show any signs of increased oxidative stress or increased susceptibility to oxidative stress-induced damage in the brain. Deletion of xCT does result in decreased extracellular glutamate concentrations in the hippocampus and striatum [33, 34]. Genotypes were confirmed by PCR amplification of ear DNA using REDExtract-N-Amp Tissue PCR Kit (Sigma-Aldrich, USA) and the following primers: 5′-GATGCCCTTCAGCTCGATGCGGTTCACCAG-3′ (GFPR3); 5′-CAGAGCAGCCCTAAGGCACTTTCC-3′ [mxCT5′flankF6]; 5′-CCGATGACGCTGCCGATGATGATGG-3′ [mxCT(Dr4)R8].

As for the BM transplanted mice, conventional chimeras were generated as described previously [50, 51], with the following modifications: recipients were C57BL/6J mice and to obtain conventional BM chimera, donor cells were derived from xCT^−/−^ mice and xCT^+/+^ littermate controls. In brief, recipient animals were sublethally irradiated with 1100 cGy (split dose). Donor animals were sacrificed by cervical dislocation. BM cells were isolated by flushing of the femur and tibia, resuspended and MACS®-depleted of CD90^+^ T cells. Recipients were reconstituted with 8–16 × 10^6^ donor BM cells 8 h after irradiation. Mice were kept on 0.01% Enrofloxacin (Baytril®, Bayer Health Care) in drinking water for 4 weeks. Engraftment took place over 8 weeks of recovery.

### Induction and assessment of EAE

EAE was induced in C57BL/6J, xCT^−/−^, and xCT^+/+^ mice as well as BM-transplanted mice using the Hooke Laboratories kit EK-2110 (Hooke Laboratories, Inc., USA) and according to the manufacturer’s instructions. Sham-treated mice received an injection of adjuvant components that was identical for the study group with omission of specific antigens (Hooke Laboratories kit CK-7110, Inc., USA). Briefly, mice were immunized by subcutaneous injection of a (MOG)_35–55_ emulsion in complete Freund’s adjuvant, followed by an intraperitoneal injection with pertussis toxin in phosphate-buffered saline (PBS) immediately after immunization as well as 24 h later. Starting from day 7 post-immunization, animals were daily evaluated for clinical signs of disease, according to the following scoring system: 0, no clinical/obvious changes; 0.5, the tip of the tail is limp; 1, limp tail; 1.5, limp tail and hind leg inhibition; 2, limp tail and weakness of the hind legs; 2.5, limp tail and dragging of hind legs; 3, limp tail and complete paralysis of the hind legs; 3.5, limp tail and complete paralysis of the hind legs in addition to unable to right itself when placed on its side; 4, limp tail, complete hind leg and partial front leg paralysis; 4.5, complete hind and partial front leg paralysis, no movement around the cage, mouse is not alert; and 5, moribund or dead. According to the ethical guidelines for animal experimentation, mice with a clinical score of 4 or higher for two consecutive days were excluded from the experiments. A researcher blinded to the genotype performed all scorings and area under the curve (AUC), and highest clinical score reached were analyzed and compared between genotypes.

C57BL/6J mice were sacrificed 25 days after EAE induction by cervical dislocation. The brain, spinal cord, and spleen were collected, snap frozen, and stored at −80 °C until use. xCT^+/+^ and xCT^−/−^ mice were sacrificed with an overdose of pentobarbital (Nembutal®; Ceva Sante Animale, Brussels, Belgium) 25 days after EAE induction, followed by transcardial perfusion with saline and collection of the spinal cord for further histologic examination. BM-transplanted mice were sacrificed 15 days following EAE induction and transcardially perfused with saline (histological analysis) or ice-cold PBS (FACS analysis).

### Histology

Mouse tissue was postfixed in 4% paraformaldehyde in 0.1 M PBS overnight, transferred to a 10% sucrose solution in 0.1 M PBS for 24 h and preserved in a 25% sucrose solution in 0.1 M PBS at 4 °C. A 15-mm section of the lumbosacral spinal cord was placed in Tissue Tek OCT compound (Sakura, The Netherlands) and frozen on dry ice. Next, the tissue was sliced on a cryostat in 20-μm sections that were mounted on Superfrost Ultra Plus slides (Thermo Scientific, Waltham, MA, USA) and stored at −80 °C until further analysis. To determine the extent of demyelination, luxol fast blue (LFB) staining was performed. Slides were immersed in 0.1% LFB solution (0.5 g LFB, 500 ml 99% ethyl alcohol, 2.5 ml glacial acetic acid) for 16 h at 56 °C. Differentiation was performed in 0.05% lithium carbonate solution. Afterwards, the slides were gradually dehydrated in increasing concentrations of ethyl alcohol followed by xylene and coverslipped with DPX mounting medium (VWR, Radnor, PA, USA). Histological quantification was done by defining a demyelination score: 0, normal white matter; 1, rare foci; 2, a few areas of demyelination; and 3, large areas of demyelination.

The amount of invading cells was analyzed by cresyl violet staining using standard staining procedures. The sections were scored for infiltration: 0, no infiltrating cells; 1, few infiltrating cells; 2, numerous infiltrating cells; and 3, widespread infiltration. Scoring was performed blinded.

### Iba1 and MBP immunofluorescence

Ionized calcium-binding adapter molecule 1 (Iba1) and myelin basic protein (MBP) immunohistochemistry was performed to visualize respectively microglial cells and the myelin status of the spinal cord. Twenty micrometers of frozen sections were permeabilized in 1% Triton X/PBS for 5 min and incubated with the primary antibodies rat anti-MBP (1:500, Merck Millipore, Darmstadt, Germany) or rabbit anti-Iba1 (1:500, Wako, Neuss, Germany) at 4 °C overnight. Cy3-conjugated goat anti-rat or goat anti-rabbit (1:500, Merck Millipore, Darmstadt, Germany) were used as secondary antibodies, and cell nuclei were stained by DAPI (Invitrogen, Carlsbad, USA). After immunostaining, images were acquired with a fluorescence microscope (BX51; Olympus, Tokyo, Japan) equipped with a digital camera (Colorview III, Olympus, Tokyo, Japan). Demyelination (MBP score) was assessed blindly using the following scoring system: 1, no demyelination; 2, rare foci; 3, few areas of demyelination; and 4, confluent areas of demyelination. Microglial activation (Iba1 score) was evaluated blindly using the following system: 1, no activation; 2, mild activation; 3, strong activation; and 4, massive activation.

### Real-time PCR

Total RNA was isolated from scrapings of the 50 μm slices of the human tissue using the TRIzol® (Life Technologies, USA) extraction method. cDNA was generated by the Taqman reverse transcription kit (Life Technologies, USA), and SYBR®Green Real-Time-PCR Master Mix (Life Technologies, USA) was used to perform real-time PCR. xCT RNA expression was analyzed using primer sets designed with the Primer Express 3 software (Applied Biosystems®, Life Technologies, USA): xCT upstream primer 5′-TGATTCATGTCCGCAAGCA-3′ and downstream primer 5′-TGTCGAGGTCTCCAGAGAAGAG-3′ and the reference gene GAPDH upstream primer 5′-TGCACCACCAACTGCTTAGC-3′ and downstream primer 5′-GGCATGGACTGTGGTCATGAG-3′. All primer sets had efficiencies between 98 and 100% over 5 log changes and amplified a single band as determined by melting curve analysis.

### Western blot analysis

Human NAWM samples and mouse CNS tissue (brain and spinal cord) were homogenized in ice-cold extraction buffer [2% sodium dodecyl sulfate (SDS), 60 mM Tris base, pH 6.8, 100 mM dithiothreitol, and 1 mM ethylenediaminetetraacetic acid] containing 1 mM sodium orthovanadate and 1% phosphatase inhibitor cocktail 3 (Sigma-Aldrich, USA). Next, samples were incubated for 30 min at 37 °C, followed by centrifugation for 10 min at 10,000×*g*. Mouse spleen tissue was homogenized in ice-cold RIPA buffer [150 mM sodium chloride (NaCl), 1% Triton X-100, 0.5% sodium deoxycholate, 0.1% SDS and 50 mM Tris, pH 8.0, 1% phosphatase inhibitor cocktail]. Samples were incubated for 5 min at 100 °C, followed by centrifugation for 30 min at 10,000×*g*. Supernatants from all homogenates were stored in aliquots at −20 °C.

Protein concentrations were quantified using the NanoDrop Spectrophotometer ND-1000 (Thermo Scientific, Waltham, MA, USA). Equal concentrations of proteins were loaded on the gel. Following separation of proteins by SDS-polyacrylamide gel electrophoresis (PAGE; 4–12% Bis-Tris precast gel, Bio-Rad Laboratories, USA) under reducing conditions, proteins were transferred to a polyvinylidene fluoride membrane (Immun-Blot PVDF Membrane, Bio-Rad Laboratories, USA). After 1-h incubation with 5% membrane blocking agent (RPN2125V; GE Healthcare, UK), blots were incubated overnight at 4 °C with an immunoaffinity purified rabbit polyclonal antibody to xCT diluted in membrane blocking agent (NB300-318, lot number G3; Novus Biologicals, USA; 1/2000 for NAWM and mouse spleen; 1/5000 for the mouse brain and spinal cord) [52]. Next, the membranes were incubated with horseradish peroxidase (HRP)-conjugated anti-rabbit immunoglobulin G antiserum (1/4000 for NAWM; 1/15000 for mouse CNS homogenates and spleen; 30 min; Dako, Denmark). Immunoreactive bands were visualized using enhanced chemiluminescence (ECL Select, RPN2235; GE Healthcare, UK) and ImageQuant™ LAS 4000 biomolecular imager (GE Healthcare) and analyzed by ImageJ software (National Institute of Health, Bethesda, MD, USA) to obtain the optical densities (OD). OD of the immunoreactive bands was normalized to the OD of the entire lane after a total protein stain (SERVA purple; SERVA Electrophoresis GmbH, Germany). An arbitrarily chosen control sample was set as a reference (100%), and the OD of all other samples was calculated relative to this reference. As molecular weight standard, Novex Sharp Protein Standard (LC5800; Novex by Life Technologies, USA) was used. The specificity of the xCT antibody was confirmed on the knockout tissue [52].

For the detection of inducible nitric oxide synthase (iNOS), nuclear factor-κB (NF-kB), phosphorylated NF-kB (p-NF-kB), glycogen synthase kinase 3β (GSK3β), phosphorylated GSK3β (p-GSK3β), Akt, phosphorylated Akt (p-Akt), eukaryotic initiation factor 2α (eIF2α), phosphorylated eIF2α (p-eIF2α), and ATF4, human brain samples were prepared and separated by SDS-PAGE as described above. Proteins were transferred to a nitrocellulose membrane, and the membranes were blocked with 5% skim milk in TBS-T (20 mM Tris buffer, pH 7.5, 0.5 M NaCl, 0.1% Tween 20) for 2 h at room temperature and incubated overnight at 4 °C in the primary antibody diluted in 5% BSA in TBS/0.05% Tween 20. The primary antibodies and dilutions were mouse anti-p-NF-kB p65 (Ser536) (#3036, 1/1000), rabbit p-GSK3β (Ser21/9) (#9331, 1/1000), rabbit p-Akt (Ser473) (#9271, 1/1000), rabbit p-eIF2a (Ser51) (#9721, 1/1000) and mouse eIF2a (#2103, 1/1000) and rabbit anti-actin (#5125, 1/20000) from Cell Signaling, USA; mouse anti-iNOS (#610431, 1/1000) and mouse GSK3β (#G22320; 1/1000) from BD Transduction Labs, USA; mouse Akt (#05-591, 1/1000) from Millipore; and rabbit anti-ATF4 (#sc-200, 1/500) and rabbit anti-NF-kB p65 (#sc-372, 1/500) from Santa Cruz Biotechnology, USA. Blots were washed in TBS/0.05% Tween 20 and incubated for 1 h at room temperature with an appropriate HRP-coupled secondary antibody. After additional washing, protein bands were detected by chemiluminescence using the Super Signal West Pico Substrate (Pierce, USA). For relative NF-kB phosphorylation, the blots were sequentially probed with phospho- and total protein antibodies. For all other antibodies, the same membrane was re-probed for actin. Autoradiographs were scanned using a Bio-Rad GS-800 scanner. Band density was measured using the manufacturer’s software. Relative NF-kB, GSK3β, eIF2α, and Akt phosphorylation was calculated as the ratio of band densities obtained by the phospho-specific antibody and the antiserum recognizing the same protein irrespective of its phosphorylation state. Relative ATF4 and iNOS expression was normalized to actin band density.

### FACS analysis

For immune cell isolation from the CNS, lethally anesthetized animals were transcardially perfused with ice-cold PBS. The brain and spinal cord were isolated, cut into small pieces, and diluted in Iscove’s modified Dulbecco’s medium (IMDM; Life Technologies, USA) substituted with 5 mg/50 μL collagenase/clostridiopeptidase (Sigma-Aldrich, USA), 1 mg/ml collagenase/dispase (Roche, Germany), and 1000 U/50 μL DNAse (Roche, Germany). After incubation for 30 min at 37 °C in the water bath, the CNS tissue was put through a mesh (100 μm) and mononuclear cells were separated by conventional 40/70 Percoll centrifugation. FACS analysis of surface markers was performed directly after isolation. Cytokine analysis was preceded by plate-bound anti-CD3/anti-CD28 stimulation for 4 h (brefeldin A was added after 2 h). CNS cells were pre-gated on lymphocytes and monocytes, followed by selection of CD45 high cells. Antibodies used for FACS surface and intracellular stainings are as follows: CD45-efluor 605 (eBioscience, USA), CD4-V450 (BD Biosciences, USA), CD45R-AlexaFluor 700 (eBioscience, USA), CD11b-Pe Cy7 (BD Biosciences, USA), IAb-PE (BD Biosciences, USA), IL-17-PE (BD Biosciences, USA), IFN-γ-AlexaFluor 700 IL-17-PE (BD Biosciences, USA), and FoxP3-APC (eBioscience, USA). Cells were run on a FACS Canto II (BD Biosciences, USA).

### Statistical analysis

Statistical analysis was performed using GraphPad Prism 6.0 software. Data are expressed as mean ± standard error of the mean (SEM). Significance was assessed by Mann-Whitney *U* test. Differences between groups were rated significant at *p* < 0.05. Correlations between xCT protein and ATF4/p-NF-κB(p65)/NF-κB(p65)/iNOS protein expression levels were computed using Pearson’s test.

## Results

### xCT protein levels are increased in the NAWM of MS patients

Characteristics of MS patients and controls without neurologic disease that were included in this study are outlined in Table 1. There is a significant difference in age and postmortem delay between the group of controls and MS patients; however, there is no correlation between those two parameters and xCT protein levels or any other protein evaluated in these samples (results not shown).

**Table 1:** Features of NAWM tissue from MS patients and controls

	Sample reference	Gender	Age	Structure	Postmortem delay (hours)
Controls	3204	M	80	NAWM, frontal	11
	3529	M	58	NAWM, frontal	9
	3531	M	74	NAWM, frontal	13.6
	3540	M	68	NAWM, frontal	10.5
	3543	F	73	NAWM, frontal	12
MS patients	2946	M	59	Adjacent NAWM, parietal	15
	3010	F	50	Adjacent NAWM, parietal	15
	3161	F	51	Adjacent NAWM, parietal	19.5
	3163	F	57	Adjacent NAWM, frontal	19.8
	3185	M	50	Adjacent NAWM, cerebellum	13.8
	3816	F	47	Adjacent NAWM, frontal	20.7
	3867	M	75	Adjacent NAWM, parietal	12.9
	4107	F	52	Adjacent NAWM, frontal	20.6

*F* female, *M* male, *MS* multiple sclerosis, *NAWM* normal appearing white matter; sample reference, numbers given by Human Brain and Spinal Fluid Resource Center in Los Angeles

**Fig. 1:**
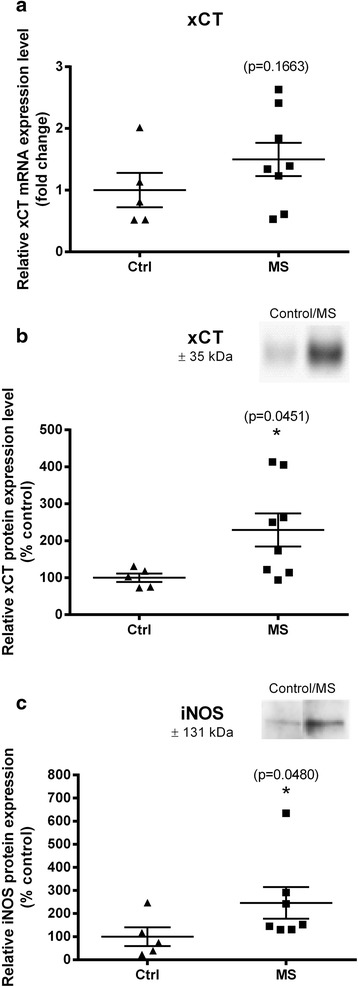
Increased xCT and iNOS protein expression in the NAWM of MS patients. **a** Real-time PCR revealed no significant difference in xCT mRNA levels between the NAWM of MS patients and corresponding tissue of control subjects when normalized to GAPDH as internal control. xCT (**b**) and iNOS (**c**) protein levels are significantly increased in NAWM of MS patients compared to controls as measured using semi-quantitative Western blotting. Normalization was performed by using total protein staining. Individual expression levels are presented as scatter dot plot and horizontal lines represent mean ± SEM (Mann-Whitney *U* test, *p < 0.05). Controls (*n* = 5), MS patients (*n* = 8). Protein bands for xCT (**b**) and iNOS (**c**) of a representative sample of control and MS patients are shown in the *inset*

No significant differences in xCT mRNA levels were observed between NAWM of MS patients (*n* = 8) and controls (*n* = 5), (fold change controls vs. MS patients: 1.00 ± 0.28 vs. 1.50 ± 0.27; *p* = 0.166, Fig. 1a). However, at the protein level, xCT expression was significantly upregulated in NAWM of MS patients compared to controls, as measured using semi-quantitative Western blotting (relative expression controls vs. MS patients: 100.00 ± 11.51 vs. 229.30 ± 44.81%; *p* = 0.045, Fig. 1b).

Recently, a pathway via Akt, GSK3β, eIF2, and ATF4 has been described to upregulate xCT expression [53]. In addition, many inflammatory stimuli that activate NF-κB-mediated transcription have been reported to activate the transcription of xCT [36, 37, 54, 55]. Therefore, we studied the protein levels of ATF4, pGSK3β, p-Akt, and p-eIF2α by Western blotting. Expression levels of p-Akt, p-eIF2α, and p-GSK3β were too faint to be quantified in both groups (results not shown). ATF4 relative expression levels were not statistically different between controls and MS patients (100.00 ± 15.14 vs. 162.80 ± 49.46; *p* = 1.00; *n* = 5–8, Table 2). Although no change in activation of NF-κB was observed in NAWM of MS patients, as reflected by unaltered relative expression levels of p-NF-κB p65 [56] compared to total p65 (relative expression controls vs. MS patients: 100.00 ± 10.54 vs. 141.40 ± 32.55%; *p* = 1.00; *n* = 5–8, Table 2), we could detect increased expression levels of iNOS, known to be induced by NF-κB [57] (relative expression controls vs. MS patients: 100.00 ± 40.25 vs. 246.60 ± 68.74%; *p* = 0.048; *n* = 5–7, Fig. 1c).

**Table 2:** Relative expression values of proteins possibly involved in regulating xCT transcription and xCT expression in NAWM samples of controls and MS patients

	Sample reference	xCT*	ATF4/actin	p-GSK3β	p-NF-κB(p65)/NF-κB	NF-κB(p65)/actin	iNOS/actin*
Controls	3204	0.73	0.396	^−^	0.334	0.782	0.037
	3529	1.18	0.714	+	0.533	0.954	0.069
	3531	1.31	1.050	^−^	0.378	1.060	0.021
	3540	0.75	0.959	^−^	0.299	3.390	0.110
	3543	1.03	1.05	^−^	0.358	1.090	0.232
MS patients	2946	2.63	0.972	^−^	0.291	0.952	0.273
	3010	4.05	3.590	++	0.979	0.520	0.136
	3161	0.94	0.914	+	0.266	2.140	0.227
	3163	2.50	0.668	^−^	0.317	0.950	0.595
	3185	1.22	2.810	^−^	0.286	1.600	1.310
	3816	1.73	0.726	+	1.166	0.491	0.143
	3867	4.13	0.616	^−^	0.409	0.856	0.123
	4107	1.14	0.553	^−^	0.581	0.777	0.122

*ATF4* activating transcription factor 4, *iNOS* nitric oxide synthase, *MS* multiple sclerosis, *NAWM* normal appearing white matter, *NF*-*κB* nuclear factor-κB, *p*-*GSK3β* phosphorylated glycogen synthase kinase 3β, *p*-*NF*-*κB* phosphorylated nuclear factor-κB; sample reference, numbers given by Human Brain and Spinal Fluid Resource Center in Los Angeles

*comparison between control and MS patients, Mann-Whitney *U *test, *p* < 0.05

**Fig. 2:**
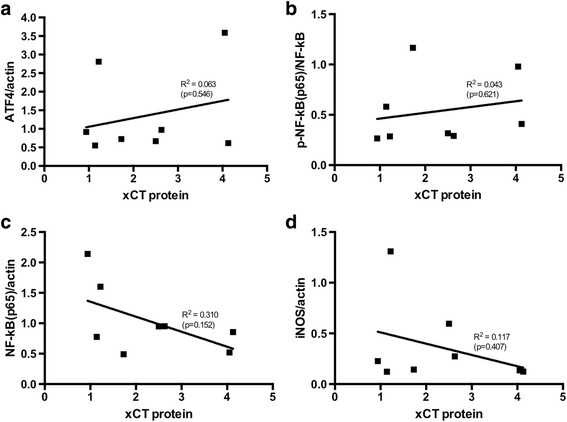
Correlation graphs show no significant correlation between proteins that regulate xCT transcription and xCT protein expression in NAWM samples of controls and MS patients for **a** ATF4, **b** p-NF-κB(p65), **c** NF-κB(p65), and **d** iNOS (Pearson’s test). *Axes* indicate relative expression levels as indicated in Table 2

Since variability between samples is inherent to the use of human material and can affect statistical analysis, we correlated expression levels of xCT to expression of ATF4 as well as NF-κB p65 and iNOS. No significant correlations were identified for ATF4 (*R* ^2^ = 0.063, *p* = 0.546; Fig. 2a), p-NF-κB(p65)/NF-κB (*R* ^2^ = 0.043, *p* = 0.621; Fig. 2b), NF-κB(p65)/actin (*R* ^2^ = 0.310, *p* = 0.152; Fig. 2c), or iNOS (*R* ^2^ = 0.117, *p* = 0.407; Fig. 2d).

In conclusion, although xCT expression levels were increased in NAWM of MS patients compared to controls, we could neither identify the pathway that leads to this upregulation nor correlate the changes in xCT expression with inflammatory markers.

### xCT protein expression levels are increased in the CNS and spleen of C57BL/6J mice after EAE induction

**Fig. 3:**
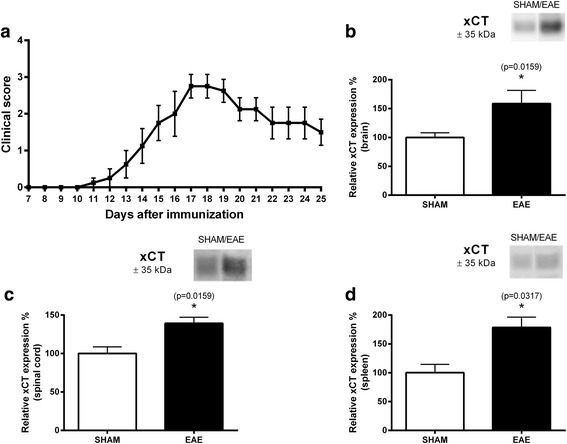
xCT protein expression is increased in the CNS and spleen of C57BL/6J mice after EAE induction. **a** Clinical score of C57BL/6J mice after EAE induction (*n* = 4). Sham-treated mice (*n* = 5) were included in this study to correct for adjuvant-induced responses and had a clinical score of 0 throughout the entire experiment. xCT protein expression levels in the brain (**b**), spinal cord (**c**), and spleen (**d**) were significantly increased in C57BL/6J mice after EAE induction compared to sham-treated animals as measured using semi-quantitative Western blotting (data are expressed as the mean ± SEM). Mann-Whitney *U* test, *p < 0.05. A representative xCT band for the brain (**b**), spinal cord (**c**), and spleen (**d**) of control and EAE mice is shown in the inset

To corroborate our findings and clarify their functional significance, we first studied xCT protein expression levels in the brain, spinal cord, and spleen of EAE-induced C57BL/6J mice (*n* = 4) compared to sham-treated mice (*n* = 5) that only received adjuvant components. Clinical signs were established from day 7 after immunization with an increase of severity until day 17, followed by partial recovery (Fig. 3a). A significant increase in xCT protein expression at day 25 was observed in the brain (relative expression control mice vs. EAE mice: 100.00 ± 8.17 vs. 158.80 ± 22.76%; *p* = 0.016, Fig. 3b), spinal cord (relative expression control mice vs. EAE mice: 100.00 ± 8.55 vs. 139.50 ± 7.78%; *p* = 0.016, Fig. 3c), and spleen (relative expression control mice vs. EAE mice: 100.00 ± 14.31 vs. 178.50 ± 17.79%; *p* = 0.032, Fig. 3d) of EAE-induced mice compared to sham controls.

### xCT^−/−^ mice are not protected against EAE development compared to xCT^+/+^ mice

**Fig. 4:**
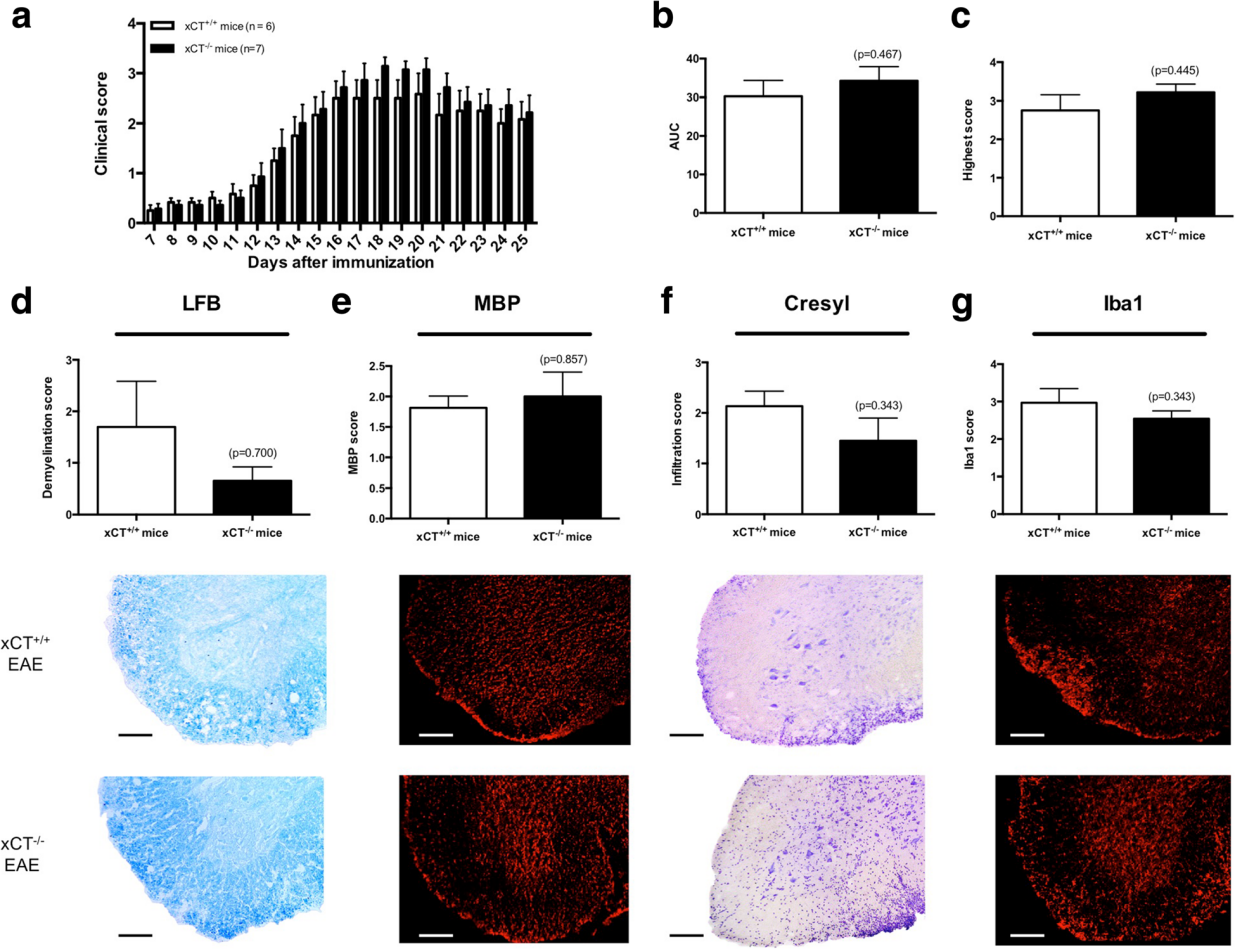
xCT^−/−^ and xCT^+/+^ mice are equally susceptible for EAE development. Clinical score of xCT^+/+^ (*n* = 6) and xCT^−/−^ mice (*n* = 7) after EAE induction (**a**). No significant differences in EAE severity were found between the two genotypes comparing AUC (**b**) and highest score reached (**c**). On day 25, the spinal cord was collected, sliced, and stained for demyelination (**d**, **e**), infiltration (**f**), and microglial activation (**g**) and subsequently scored (*n* = 3-4). Scoring was performed blinded. Data are expressed as mean ± SEM, Mann-Whitney *U* test. Representative images of LFB (**d**), MBP (**e**), cresyl violet (**f**), and Iba1 (**g**) staining in the lumbar section of EAE-induced xCT^+/+^ and xCT^−/−^ mice are shown below each graph (scale bar, 100 μm)

There was no difference in clinical outcome of xCT^−/−^ mice and their xCT^+/+^ littermates in the EAE model (Fig. 4a), as reflected by similar areas under the curve (AUC; xCT^+/+^ vs. xCT^−/−^ mice: 12.38 ± 1.74 vs. 14.46 ± 1.21; *p* = 0.467; *n* = 6–7; Fig. 4b) and highest score reached (xCT^+/+^ vs. xCT^−/−^ mice: 2.75 ± 0.40 vs. 3.21 ± 0.21; *p* = 0.445; *n* = 6–7; Fig. 4c).

To compare the histopathological hallmarks after EAE induction between xCT^+/+^ and xCT^−/−^ mice, LFB, MBP, cresyl violet, and Iba1 stainings were performed 25 days post-immunization to visualize demyelination (LFB, MBP), infiltration of cells (cresyl violet), and microglial activation (Iba1) (for representative slices, see Fig. 4d–g). No significant differences in demyelination (LFB demyelination score xCT^+/+^ vs. xCT^−/−^ mice: 1.70 ± 0.89 vs. 0.66 ± 0.26; *p* = 0.700; *n* = 3; Fig. 4d; MBP score xCT^+/+^ vs. xCT^−/−^ mice: 1.81 ± 0.19 vs. 2.00 ± 0.40; *p* = 0.857; *n* = 3–4; Fig. 4e) or infiltration (infiltration score xCT^+/+^ vs. xCT^−/−^ mice: 2.14 ± 0.30 vs. 1.45 ± 0.45; *p* = 0.343; *n* = 3–4; Fig. 4f) scores were found between the two groups. In addition, no significant alterations in microglial activation between xCT^+/+^ and xCT^−/−^ mice were detected (Iba1 score xCT^+/+^ vs. xCT^−/−^ mice: 2.97 ± 0.38 vs. 2.54 ± 0.20; *p* = 0.343; *n* = 3–4; Fig. 4g).

### Mice lacking xCT in immune cells are less sensitive to EAE disease than wild-type control mice

**Fig. 5:**
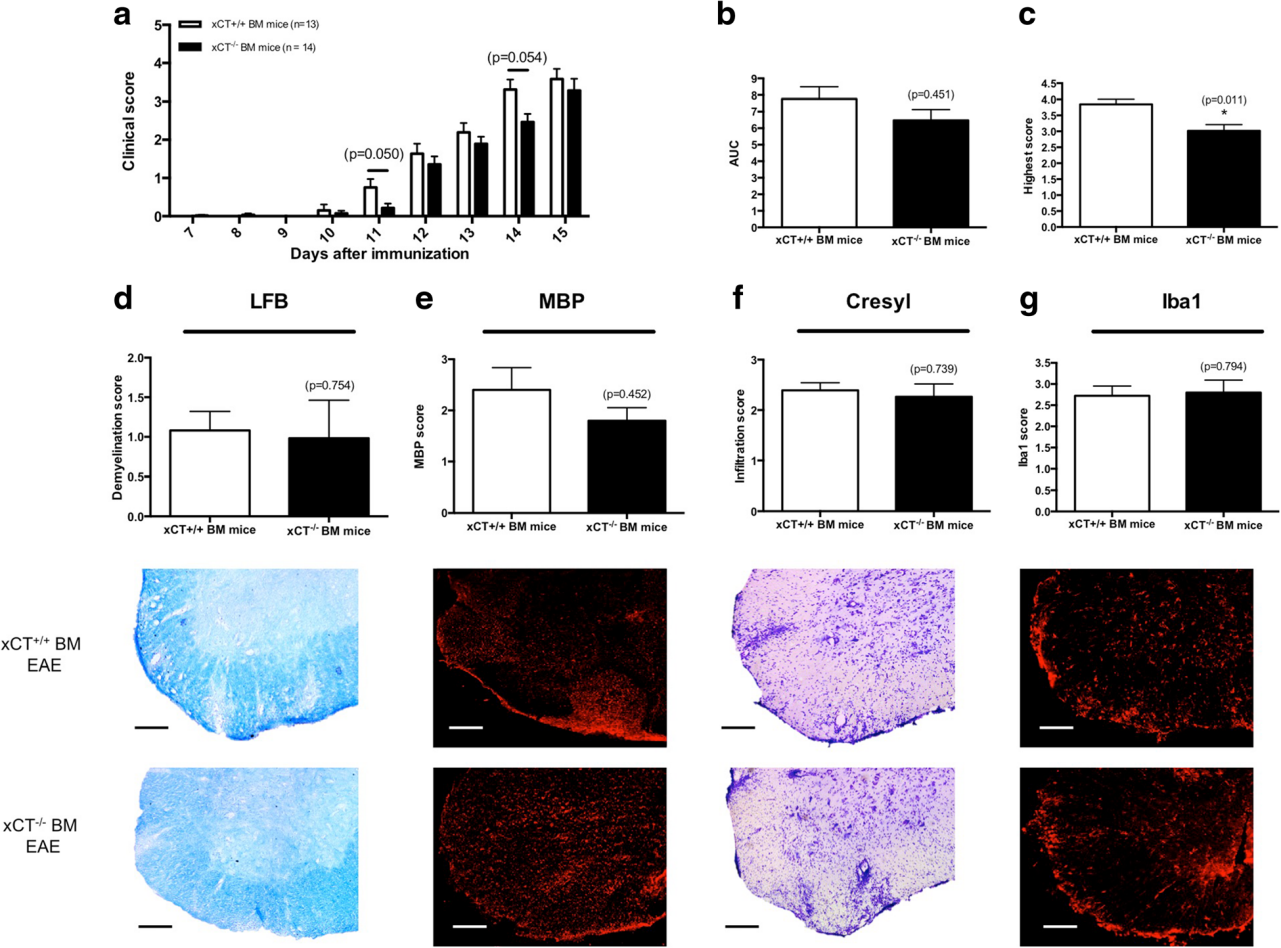
Mice lacking xCT in immune cells are less sensitive to EAE disease than mice expressing xCT in immune cells. Clinical scores of BM-transplanted mice expressing (*n* = 13) or lacking (*n* = 14) xCT in their immune cells are different 11 and 14 days after EAE induction (**a**), as well as highest score reached (**c**). No differences were observed in AUC (**b**). On day 15, the spinal cord was collected, sliced, and stained for demyelination (**d**, **e**), infiltration (**f**), and microglial activation (**g**) and subsequently scored (*n* = 4–5). Scoring was performed blinded. Data are expressed as mean ± SEM, Mann-Whitney *U* test, *p < 0.05. Representative images of LFB (**d**), MBP (**e**), cresyl violet (**f**), and Iba1 (**g**) staining of the lumbar section of EAE-induced mice transplanted with xCT^+/+^ or xCT^−/−^ BM are shown below each graph (scale bar, 100 μm)

To differentiate between the effects of system x_c_^−^ on immune cells invading the CNS versus the resident cells of the CNS, we collected BM from both xCT^+/+^ and xCT^−/−^ mice and transplanted it into irradiated xCT^+/+^ mice, as such generating mice with a system x_c_^−^ deficiency only in the immune system. Due to the rapid progress of EAE severity in these BM-transplanted mice, they had to be sacrificed at day 15 according to animal welfare restrictions. Although no differences in AUC were assessed between genotypes (xCT^+/+^ vs. xCT^−/−^ BM mice: 7.77 ± 0.73 vs. 6.47 ± 0.66; *p* = 0.451; *n* = 13–14; Fig. 5b), a strong trend for a significant difference in EAE clinical score could be detected 11 (xCT^+/+^ vs. xCT^−/−^ BM mice: 0.75 ± 0.23 vs. 0.21 ± 0.12; *p* = 0.050; *n* = 13–14) and 14 days (xCT^+/+^ vs. xCT^−/−^ BM mice: 3.31 ± 0.26 vs. 2.46 ± 0.21; *p* = 0.054; *n* = 13–14) after immunization, with a better outcome for mice lacking xCT in their immune cells (Fig. 5a). Moreover, the highest score reached by mice lacking functional xCT compared to mice expressing it on their immune cells was significantly lower (xCT^+/+^ vs. xCT^−/−^ BM mice: 3.85 ± 0.58 vs. 3.02 ± 0.73; *p* = 0.011; *n* = 13–14; Fig. 5c).

The differences in the EAE score between the two groups were not reflected in a difference in demyelination (LFB demyelination score xCT^+/+^ vs. xCT^−/−^ BM mice: 1.08 ± 0.24 vs. 0.98 ± 0.48; *p* = 0.754; *n* = 5–4; Fig. 5d; MBP score xCT^+/+^ vs. xCT^−/−^ BM mice: 2.40 ± 0.44 vs. 1.80 ± 0.26; *p* = 0.452; *n* = 5; Fig. 5e) or infiltration (infiltration score xCT^+/+^ vs. xCT^−/−^ BM mice: 2.39 ± 0.15 vs. 2.26 ± 0.25; *p* = 0.739; *n* = 5; Fig. 5f) scores as evaluated by histological staining (representative slices, see Fig. 5d–f). Furthermore, no significant differences in Iba1 levels (Iba1 score xCT^+/+^ vs. xCT^−/−^ BM mice: 2.72 ± 0.23 vs. 2.80 ± 0.29; *p* = 0.794; *n* = 5; Fig. 5g) were observed.

**Fig. 6:**
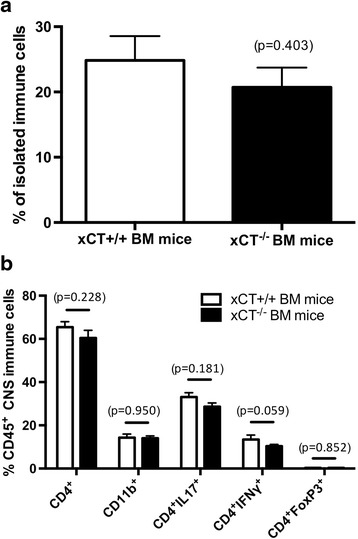
Immune cell infiltration into the CNS of irradiated mice transplanted with BM originating from xCT^+/+^ or xCT^−/−^ mice is not different between the two groups after EAE induction. Proportion of immune cells relative to the total amount of isolated cells (**a**) and the proportion of CD4^+^, CD11b^+^, CD4^+^IL-17^+^, CD4^+^IFN-γ^+^, and CD4^+^FoxP3^+^ cells within the immune cell population (**b**) in the CNS after EAE induction in mice that are expressing (xCT^+/+^ BM mice) or lacking xCT in their immune cells (xCT^−/−^ BM mice). Data shown are means ± SEM; Mann-Whitney *U* test

To exclude that the differences in the EAE score were due to alterations in peripheral immune cell infiltration into the CNS as a result of system x_c_^−^ deficiency, the presence of several immune cell populations was analyzed in the CNS of both groups of mice (i.e., mice with xCT^+/+^ and xCT^−/−^ BM) 15 days after EAE immunization, using FACS analysis. Figure 6a demonstrates that a similar number of immune cells were isolated from the CNS of both groups (xCT^+/+^ vs. xCT^−/−^ BM mice: 24.83 ± 3.72 vs. 20.71 ± 3.03; *p* = 0.403; *n* = 6–8; Fig. 6a). Moreover, the overall numbers of CD4^+^ infiltrating T cells in the CNS of BM transplanted mice were equal between genotypes (xCT^+/+^ vs. xCT^−/−^BM mice: 65.43 ± 2.52 vs. 60.44 ± 3.51%; *p* = 0.228; *n* = 6–8; Fig. 6b), as well as the proportion of infiltrating CD11b^+^ (xCT^+/+^ vs. xCT^−/−^BM mice: 14.30 ± 1.69 vs. 14.01 ± 1.07%; *p* = 0.950; *n* = 6–8; Fig. 6b), CD4^+^IL17^+^^+^ (xCT^+/+^ vs. xCT^−/−^BM mice: 33.08 ± 2.05 vs. 28.63 ± 1.75%; *p* = 0.181; *n* = 6–8; Fig. 6b), CD4^+^IFN-γ^+^^+^ (xCT^+/+^ vs. xCT^−/−^BM mice: 13.40 ± 2.13 vs. 10.40 ± 0.78%; *p* = 0.059; *n* = 6–8; Fig. 6b) and CD4^+^FoxP3^+^ (xCT^+/+^ vs. xCT^−/−^ BM mice: 0.32 ± 0.09 vs. 0.29 ± 0.04%; *p* = 0.852; *n* = 6–8; Fig. 6b) cells was identical in mice harboring xCT^+/+^ and xCT^−/−^ immune cells.

These data illustrate that the absence of xCT on immune cells decreases susceptibility for EAE induction, without affecting demyelination or infiltration of immune cells into the CNS.

## Discussion

The goal of this study was to further investigate the potential of system x_c_^−^ as a drug target for the treatment of MS. Consistent with earlier reports [41–43], our data support that system x_c_^−^ is involved in the pathophysiology of MS. Yet, our data show that the absence of system x_c_^−^ on immune cells invading the CNS, rather than resident cells of the CNS, seems to ameliorate the clinical outcome of mice in the EAE model, and therefore suggest that enhancement of system x_c_^−^ on these invading immune cells contributes to EAE disease.

Glutamate homeostasis plays an essential role in limiting neuronal toxicity because elevated extracellular glutamate levels can lead to excessive activation of ionotropic glutamate receptors and, as a result, excitotoxicity and neuronal death. Glutamate toxicity has been shown to be a key player in several neurological disorders [8, 58]. System x_c_^−^ releases glutamate in exchange for cystine and was shown to be the major source of extracellular glutamate in several brain regions [33–35]; enhancement of this transporter may therefore induce detrimental effects via release of toxic amounts of glutamate in the extracellular space. However, by providing cysteine [59] and stimulating GSH synthesis [45, 60], system x_c_^−^ fulfills a dual role and its relevance in neurological disorders remains a matter of debate. Previous reports have shown that xCT mRNA expression is increased in postmortem optic nerve and peripheral blood cells of MS patients [41]. Accordingly, in our study, we report for the first time the increased xCT protein levels in the NAWM of MS patients. These increased levels are most likely the result of xCT upregulation on resident CNS cells rather than infiltrating immune cells given the absence of overt leukocyte infiltration in the NAWM [61]. In combination with the previously reported reduction in EAAT2 expression at the periphery of MS lesions [15, 62], glutamate release via system x_c_^−^ might lead to excitotoxicity, in accordance with elevated glutamate levels reported in NAWM of MS patients [11].

Several transcription factors that regulate xCT expression have been identified. Recently, the PI3K/Akt/GSK3β/eIF2α/ATF4 pathway was shown to be responsible for increased xCT expression in the hippocampus of patients suffering from temporal lobe epilepsy [53]. ATF4 binds to the amino acid response element in the xCT promoter region to induce xCT transcription. Moreover, the phosphorylation of eIF2α in response to cellular stress situations has been associated with other white matter disorders such as childhood ataxia with central hypomyelination/vanishing white matter disease syndromes, a collection of autosomal recessive neurological leukodystrophies that are characterized by white matter hypomyelination [63]. Elevated levels of phosphorylated eIF2α are also found in CNS oligodendrocytes of EAE mice compared to control animals [64]. In the present study, however, we could not correlate increased xCT protein expression levels in NAWM of MS patients to ATF4 alterations.

Many inflammatory stimuli, including the cytokine interleukin-1β [54] and tumor necrosis factor α [36] as well as bacterial lipopolysaccharide (LPS), which are associated with the activation of the transcription factor NF-kB [37, 55, 65] have been shown to upregulate xCT [36, 37, 54]. A classical NF-kB downstream target in the context of inflammation is iNOS [66]. Although some evidence suggests that xCT is not a direct downstream target of NF-kB [37], NF-kB and its downstream targets might represent surrogate parameters for the still unknown mechanism of inflammatory upregulation of xCT. Moreover, several studies provide evidences that NF-κB is involved in MS and EAE [67]. In line with these observations and in accordance with previous studies [67–69], we observed a significant upregulation of iNOS in NAWM of MS patients. Yet, despite the increased iNOS expression levels, no changes could be identified in either the expression of NF-κB or its phosphorylation, suggesting that iNOS expression is regulated by other signaling pathways, independent of NF-κB [70]. However, we cannot exclude that nuclear translocation of NF-κB, an essential step for activation of target genes, is the main pathway of iNOS induction in MS. Moreover, phosphorylated proteins might be subjected to dephosphorylation during the postmortem period. Still, as iNOS levels did not correlate with xCT protein levels, the pathways that regulate the expression of iNOS and xCT seem to be different in MS NAWM. As such, the transcription factor/pathway that upregulates xCT in NAWM of MS patients remains to be determined. One possibility would be Nrf2, a transcription factor that binds to an antioxidant response element in the promoter region of xCT [40]. However, it has been described that Nrf2 expression is increased around the active lesion edge in MS patients, decreasing towards the NAWM [71], and therefore, we speculate that this pathway is most likely not involved.

After EAE induction in C57BL/6J mice, we also confirmed increased xCT protein expression in the brain, spinal cord, and spleen compared to controls, in line with the observations in the spinal cord of EAE rats [41, 42]. However, Morales Pantoja et al. recently reported diminished xCT protein levels in EAE mice 21 days post-immunization; nevertheless, no differences were observed 30 days post-immunization. Since we collected our samples 25 days after EAE induction, this contradictory result may be due to the different time-point evaluated, suggesting interesting dynamics in xCT protein expression during disease course [44]. Another important reason for this contradictory finding may be the use of non-specific anti-xCT antibodies [52]: although most of the commercially available anti-xCT antibodies label a protein with a molecular weight of 55 kDa, we recently showed that xCT protein migrates at 37 kDa in SDS-PAGE and confirmed the specificity of this signal on xCT^−/−^ tissue [72].

System x_c_^−^ can fulfill a dual role in the CNS. Induction of xCT might result in an overload of glutamate into the extracellular space, supporting the hypothesis that enhancement of system x_c_^−^ during EAE disease, together with dysfunction of EAATs [18], might result in glutamate dysregulation followed by excitotoxicity, and hence axonal degeneration. On the other hand, system x_c_^−^ imports cystine in the cells, and thereby promotes the maintenance and synthesis of GSH, the most important antioxidant in the brain and essential to prevent oxidative stress and cell damage [34, 39, 73]. Several research groups demonstrated that reduction of oxidative stress ameliorates EAE [74–76], and GSH plays an important role in this process. Moreover, GSH levels are decreased in the spinal cord of EAE mice during the acute phase of the disease [77].

Surprisingly, genetic loss of xCT in C57BL/6J mice did not affect the clinical outcome after EAE induction. Nonetheless, and in contrast to our results, recent data provided by Evonuk et al. show a resistance to EAE development in sut/sut mice, which harbor a spontaneous large deletion that comprises the last exon of the *slc7a11* gene which encodes xCT and leads to functional system x_c_^−^ deficiency [43, 78]. Yet, discrepancies in EAE outcome could be due to two dissimilarities, i.e., different EAE induction protocols and the genetic background of the sut/sut mice as compared with our xCT^−/−^ mice. Sut/sut mice arose spontaneously in the C3H/HeDiSn colony, whereas xCT^−/−^ mice have a defined C57BL/6J background. Moreover, the mutation in sut/sut mice is not restricted to deletion of the last exon of *Slc7a11* but extends from intron 11 through exon 12 resulting in a truncated mRNA transcript for xCT and covers a large fragment of 480 kb [78, 79]. Sut/sut mice develop brain atrophy starting from week 15 after birth [80], a phenomenon that is not observed in xCT^−/−^ mice where no anatomical changes are found in the brain [34]. As such, results obtained in sut/sut mice are not always in line with the data from xCT^−/−^ mice [34, 79, 80]. Also, SAS was shown to attenuate EAE disease and to reduce demyelination [43]. Here, we should keep in mind that although SAS is known to inhibit system x_c_^−^ [81], it also exerts anti-inflammatory actions [82], which can obviously bias the result. Yet, the authors confirmed these results with (S)-4-carboxyphenylglycine (CPG), a group I metabotropic receptor antagonist that also inhibits system x_c_^−^ [83], supporting the idea that the observed effect is most probably related to system x_c_^−^ inhibition. Finally, it is important to mention that sut/sut mice were immunized with PLP to induce EAE, a model that is closely related to relapsing-remitting MS, whereas our xCT^−/−^ mice were EAE-induced with MOG, which serves as a more chronic model. Therefore, discrepancies in the effects of xCT deficiency on EAE severity might arise from differences in the experimental EAE paradigm, background strain or the genetic modification that leads to xCT deficiency.

In contrast to the observations described above, Soria et al. recently showed that chronic administration of SAS causes a decrease in myelin levels together with a significant reduction in rotarod performance after 28 days of treatment [45]. Despite the fact that the essential role of the cystine/glutamate antiporter for oligodentrocyte survival in vitro is clear cut, as well as for other cell types such as neurons [39], it is commonly accepted that alternative mechanisms are able to provide cyst(e)ine to cells and therefore counterbalance a GSH shortage in vivo. In our hands, chronic administration of 320 mg/kg once daily or 160 mg/kg twice daily of SAS did not induce any signs of motor deficits in xCT^+/+^ nor xCT^−/−^ mice after 28 days of treatment (unpublished observations). Yet, since information such as sex and strain of the mice used in the experiment were not reported in the aforementioned study, we cannot exclude that these discrepancies are related to these essential factors.

xCT mRNA is upregulated in peritoneal macrophages after injection with LPS [36] and MOG peptide (unpublished results), in line with the upregulation of xCT protein we observed in the spleen of EAE mice. Furthermore, leukocytes from MS patients display higher xCT mRNA levels [41]. These findings suggest that system x_c_^−^ might be enhanced on immune cells in MS and after EAE induction, whereby immune cells invade the spinal cord, resulting in increased glutamate release and ensuing damage. Indeed, it is known that the blood-brain barrier (BBB) in MS disease is disrupted and that a larger activity of ROS and inflammatory cytokines promotes the migration of immune cells into the CNS to trigger inflammation and, consequently, MS lesions [84]. To test this hypothesis, we transplanted BM of xCT^−/−^ mice into irradiated mice to create animals expressing xCT throughout their entire body, except for their immune cells. This setup demonstrated a general protective outcome for mice lacking xCT in immune cells, suggesting that system x_c_^−^ on immune cells plays an important role in the degree of EAE disease and possibly, in human disease as well. The differences in clinical score that we observed in BM-transplanted mice are not related to differences in invasion of immune cells into the CNS, since the number of infiltrating cells was similar in both groups of mice, as was seen with the infiltration score as well as with the FACS analysis. It is noteworthy to mention that in the study of Evonuk and colleagues, a decrease in the number of infiltrating CD4^+^, IFN-γ^+^, IL-17A^+^, and FoxP3^+^ T cells was noticed in the spinal cord of EAE mice after SAS treatment, suggesting a modulating role in immune cell infiltration for this compound [43]. However, although the authors also looked at the effects of CPG as well as genetic loss of xCT on the clinical outcome after EAE induction, they only examined the effect of SAS, and not CPG or genetic deletion of xCT, on immune cell infiltration. As such, the possible effect of the anti-inflammatory action of SAS on the BBB permeability and/or the infiltration of immune cells cannot be entirely excluded and might explain the discordance with our present study. Reduced xCT expression levels in the spinal cord of SAS-treated EAE mice compared to PBS-treated EAE mice [43] support indeed the idea that the anti-inflammatory actions of SAS are not to be neglected in this model.

So far, information about xCT expression on immune cells is quite limited, and the exact function of system x_c_^−^ in these cells needs further investigation. Cystine transport in human T cells was barely detected [85–88]. On the other hand, cystine/glutamate exchange in dendritic cells [88] and macrophages [89, 90] has been shown to mediate T cell activation and GSH synthesis, respectively. Moreover, increased glutamate levels released via system x_c_^−^ can affect T cells function, acting on AMPA receptors, known to be expressed by activated T cells [91] and increased in MS patients [92]. Treatment with NBQX, an AMPA receptor antagonist, ameliorates clinical EAE symptoms in both mice [93] and rats [94] without affecting lesion size or CNS inflammation, in line with our observations in xCT^−/−^ BM mice. Since xCT levels are increased in infiltrating dendritic cells and macrophages in EAE lesions [41] and glutamate released by dendritic cells is known to interact with T cells [91], we can speculate that the absence of xCT and the resulting missing interaction between toxic glutamate levels and AMPA receptors present on T cells might affect EAE, ameliorating the course of the disease without affecting lesion size and degree of CNS inflammation.

## Conclusions

Our findings confirm the involvement of system x_c_^−^ in MS although additional research is needed. Indeed, in contrast to other neurological disorders where loss of system x_c_^−^ is clearly beneficial, this is less straightforward in MS due to concurring effects in the CNS and in the immune system. We hypothesize that glutamate release via system x_c_^−^ on CNS-invading immune cells participates in excitotoxicity and contributes to MS pathology and that preventing the induction of xCT in inflammatory cells rather than inhibiting system x_c_^−^ pharmacologically might be a promising strategy to treat inflammatory CNS demyelination.

## Abbreviations

ATF4: Activating transcription factor 4
AUC: Area under the curve
BBB: Blood-brain barrier
BM: Bone marrow
CNS: Central nervous system
EAATs: Excitatory amino acid transporters
EAE: Experimental autoimmune encephalomyelitis
eIF2α: Eukaryotic initiation factor 2α
GSH: Glutathione
GSK3β: Glycogen synthase kinase 3β
HRP: Horseradish peroxidase
Iba1: Ionized calcium-binding adapter molecule 1
iNOS: Nitric oxide synthase
LFB: Luxol fast blue
LPS: Lipopolysaccharide
MBP: Myelin basic protein
MOG: Myelin oligodendrocyte glycoprotein
MS: Multiple sclerosis
NAWM: Normal appearing white matter
NF-κB: Nuclear factor-κB
OD: Optical density
PAGE: Polyacrylamide gel electrophoresis
p-Akt: Phosphorylated Akt
PBS: Phosphate-buffered saline
p-eIF2α: Phosphorylated eukaryotic initiation factor 2α
p-GSK3β: Phosphorylated glycogen synthase kinase 3β
PLP: Proteolipid protein
p-NF-κB: Phosphorylated nuclear factor-κB
ROS: Reactive oxygen species
SAS: Sulfasalazine
SDS: Sodium dodecyl sulfate
SEM: Standard error of the mean
sut/sut: C3H/HeSnJSlc7a11sut/sut
xCT^−/−^: xCT knockout
xCT^+/+^: xCT wild type
